# Homogenization of Radial Temperature by a Tungsten Sink in Sublimation Growth of 45 mm AlN Single Crystal

**DOI:** 10.3390/ma13235553

**Published:** 2020-12-06

**Authors:** Yue Yu, Botao Liu, Xia Tang, Sheng Liu, Bing Gao

**Affiliations:** The Institute of Technological Sciences, Wuhan University, Wuhan 430072, China; 2019106520025@whu.edu.cn (Y.Y.); 2018106520020@whu.edu.cn (B.L.); 2018106520022@whu.edu.cn (X.T.); victorliu@whu.edu.cn (S.L.)

**Keywords:** sublimation growth, AlN single crystal, thermal stress, numerical experiments

## Abstract

To reduce the thermal stress during the sublimation growth of 45 mm AlN single crystal, a tungsten sink was put on the top of the crucible lid. Numerical experiments showed that the radial temperature gradient was reduced due to the homogenization effect on temperature as a result of the sink. Therefore, this simple tungsten sink method has the potential to grow large-size AlN ingots with fewer cracks. It also reveals that enhancing the heat exchange of the crucible lid is an effective way to improve the quality of crystal growth.

## 1. Introduction

Aluminum nitride (AlN) is a promising material due to its wide band gap (6.28 eV), high thermal conductivity (340 W·m^−1^·K^−1^) and small lattice and thermal expansion mismatch with GaN [1]. AlN has great potential in high-power and high-frequency electronic and deep ultraviolet (UV) optoelectronic industries [2]. AlN single crystal is usually grown by the physical vapor transport (PVT) method [3]. However, AlN crystal growth is difficult, especially in large sizes, with cracks occurring frequently [4]. To reduce the probability of crack occurrence, it is important to reduce the thermal stress by homogenizing the radial temperature distribution. There are two methods to achieve this. The first is to perform experiments using trial and error; however, this method is expensive and time consuming. The second is numerical simulation and optimization, which is low cost and highly efficient [5]. 

Many groups have used the second method to improve AlN crystal growth and enhance crystal quality. Liu and Edgar [6] developed a global model for simulating AlN sublimation growth that included surface kinetics. Liu [7] and Wu [8] formulated an integrated model that considered induction heating, thermal system design, mass transport and growth kinetics. Gao et al. [9] established a fully coupled compressible flow model to study the sublimation and mass transport processes in AlN crystal growth. Wang et al. [10] studied the distribution and evolution of the total resolved shear stress in AlN single crystals. Wolfson and Mokhov [11] examined the dependence of growth rate on nitrogen pressure. Segal et al. [12] developed a model that considered the diffusive and convective transport of gases and the kinetic limitation of nitrogen adsorption/desorption on AlN surfaces. Wang and Deng [13] investigated the effect of the hot-zone structure on the temperature distribution in growth chambers. Wang and Zhang [14] investigated the effect of different temperature distributions on the growth of AlN crystals through simulation and experimentation. Liu et al. [15] focused on the crystal growth mode and the defects in AlN grown on different (0001) 6HSiC substrates and examined the effects of the substrate preparation on the growth mode and types of resultant defects. Zhuang and Edgar [16] studied AlN single-crystal growth using a microwave-heated furnace. Hartmann [17] grew freestanding AlN single crystals with c-plane areas exceeding 10 mm in diameter. Zhang [18] used SiC heterogeneous seeds to grow AlN single crystals with a diameter of 40 mm. Hu [19] assessed the correlation of the quality of AlN layers grown on SiC seeds with the growth temperature and orientation of the seeds.

Although many numerical studies have been conducted, most were focused on the growth of AlN crystals less than 25 mm in radius; there are few studies on larger-size AlN single crystals for industrial production. The advantages of numerical simulation are the simplification of the experimental process and reduction of the difficulty of the experiment. Thus, numerical simulation and optimization are ideal for the growth of large crystals. 

In this paper, numerical simulation was used to optimize the radial temperature distribution for minimizing the thermal stress of AlN crystal with a radius of 45 mm during sublimation growth. The simulation showed that a tungsten sink design placed on the top of the crucible lid can effectively improve the radial temperature distribution and reduce the thermal stress. 

## 2. Simulation

### 2.1. Geometric Model

**Figure 1:**
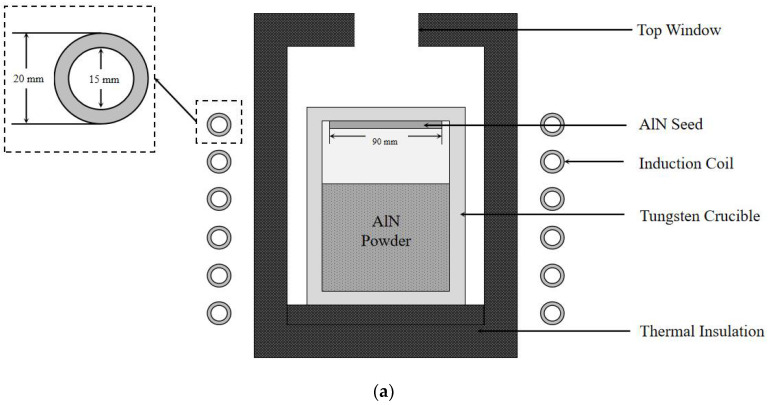

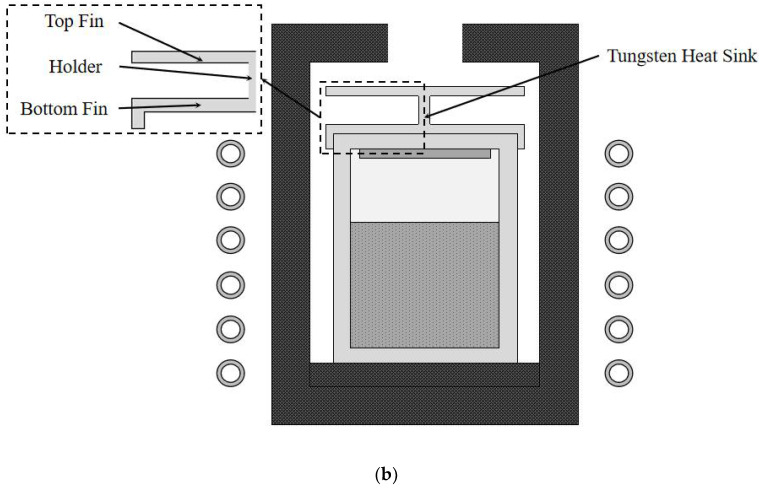
(**a**) AlN sublimation growth furnace according to traditional design; (**b**) AlN sublimation growth furnace according to improved design.

Figure 1a shows the simulated 45 mm AlN sublimation growth furnace according to traditional design. The crucible is made of tungsten and placed in a nitrogen-rich ambient. The crucible is typically 200 mm in height and 70 mm in radius. The crucible wall thickness is 20 mm. The thermal insulation is made of graphite felt. In order to simplify the calculation, the emissivity of the material is unified to 0.8. A seed 45 mm in radius and 7 mm in thickness is mounted on the bottom of the crucible lid. The growth system is heated by induction coil (10 kHz) [20,21]. Figure 1b shows the improved 45 mm growth furnace using a tungsten sink method. The sink material could also be high-density graphite with a high thermal conductivity.

### 2.2. Mathematical Model

The governing equations and the boundary conditions of heat transfer can be expressed as [20,21,22]:

(1) $$\rho C_{p}\frac{\partial T}{\partial t}=\nabla \left(k\nabla T\right)+q_{radi}+q_{eddy}$$

(2) $$\frac{q_{radi}}{\varepsilon_{j}}-\overset{N}{\underset{k=1}{\sum }}F_{j,k}\frac{1-\varepsilon_{k}}{\varepsilon_{k}}q_{radi,k}=\sigma T_{j}^{4}-\overset{N}{\underset{k=1}{\sum }}F_{j,k}\sigma T_{k}^{4}$$

(3) $$q_{eddy}=\frac{1}{2}\sigma_{c}\omega^{2}\left(A_{r}^{2}+A_{i}^{2}\right)$$

where *ρCp* is the effective heat capacity, *k* is the thermal conductivity, *q_radi_* is the radiative heat flux on the surface of the growth chamber and *q_eddy_* is the heat flux caused by the eddy current. 

Table 1 shows the thermophysical properties of materials in the growth chamber [23,24].

The thermal stress of AlN crystal is calculated according to the following equations [25,26]:

(4) $$\frac{1}{r}\frac{\partial }{\partial r}\left(r\sigma_{rr}\right)+\frac{\partial \tau_{rz}}{\partial z}-\frac{\sigma_{\phi \phi }}{r}=0$$

(5) $$\frac{1}{r}\frac{\partial }{\partial r}\left(r\tau_{rz}\right)+\frac{\partial \sigma_{zz}}{\partial z}=0$$

(6) $$\left(\begin{array}{c} \sigma_{rr}\\ \sigma_{\phi \phi }\\ \sigma_{zz}\\ \tau_{rz} \end{array}\right)=\left(\begin{array}{c} c_{11}\\ c_{12}\\ c_{13}\\ 0 \end{array}\begin{array}{c} c_{12}\\ c_{22}\\ c_{23}\\ 0 \end{array}\begin{array}{c} c_{13}\\ c_{23}\\ c_{33}\\ 0 \end{array}\begin{array}{c} 0\\ 0\\ 0\\ c_{44} \end{array}\right)\times \left(\begin{array}{c} \varepsilon_{rr}-\alpha_{r}\left(T-T_{ref}\right)\\ \varepsilon_{\phi \phi }-\alpha_{\phi }\left(T-T_{ref}\right)\\ \varepsilon_{zz}-\alpha_{z}\left(T-T_{ref}\right)\\ \varepsilon_{rz} \end{array}\right)$$

where *σ_rr_*, *σ_ΦΦ_* and *σ_zz_* represent the normal stress; *τ_rz_* represents the shear stress; *c_ij_* is the elastic constant; *ε_rr_*, *ε_ΦΦ_*, *ε_zz_* and *ε_rz_* are the strain components; *α_r_*, *α_Φ_* and *α_z_* are the thermal expansion coefficients and *T_ref_* is the reference temperature. 

The boundary conditions for thermal stress calculations are as follows [27]. The top constraint to the crystal is rigid. Because there is no contact between the crystal and crucible wall, the crystal interface is stress free. The equations are solved by the 2D finite element method.

## 3. Results and Discussion

### 3.1. Temperature Distribution

In simulations, the structure of a finned heat exchanger was used to form a tungsten sink device that was placed on the top of the crucible lid. This device consisted of two fins and a holder between them. Together, they increased the heat exchange area, which was more conducive to radiate heat to the surrounding area. To better understand the effect of the sink on temperature distribution, we set up a series of simulations.

**Figure 2:**
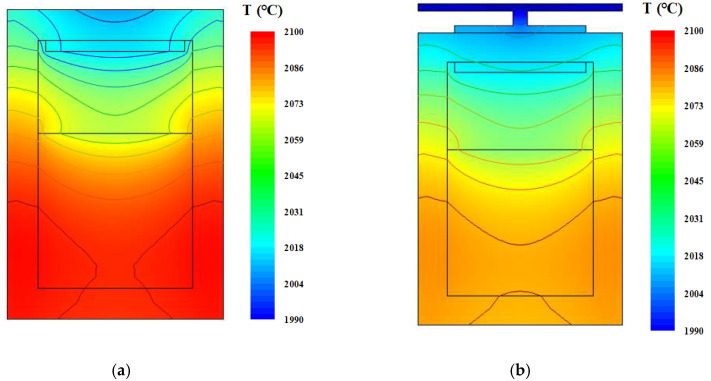

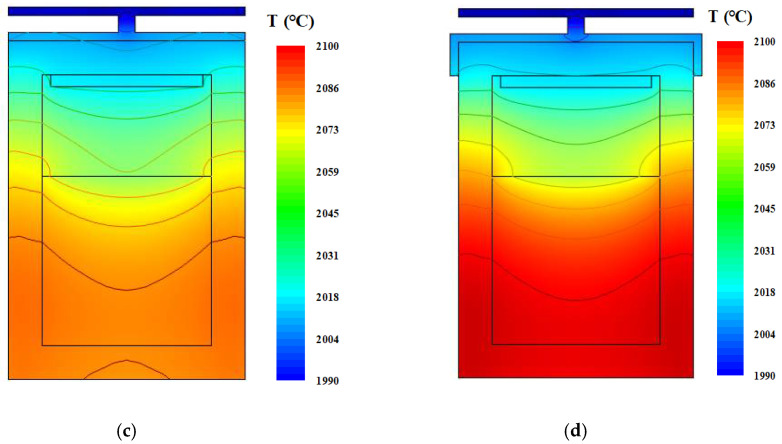
Temperature distributions for (**a**) the original design, (**b**) A design, (**c**) B design and (**d**) C design.

First, designs were chosen in which the bottom part of the tungsten sink covered the AlN seed diameter (A design), the crucible outer diameter (B design) and the whole crucible lid (C design) (see Figure 2). 

The temperature distributions for the original and three improved designs are shown in Figure 2. In all of the improved designs, the temperature distributions were more uniform in the gas chamber and near the seed. This temperature distribution is beneficial for reducing thermal stress of the crystal. 

**Figure 3:**
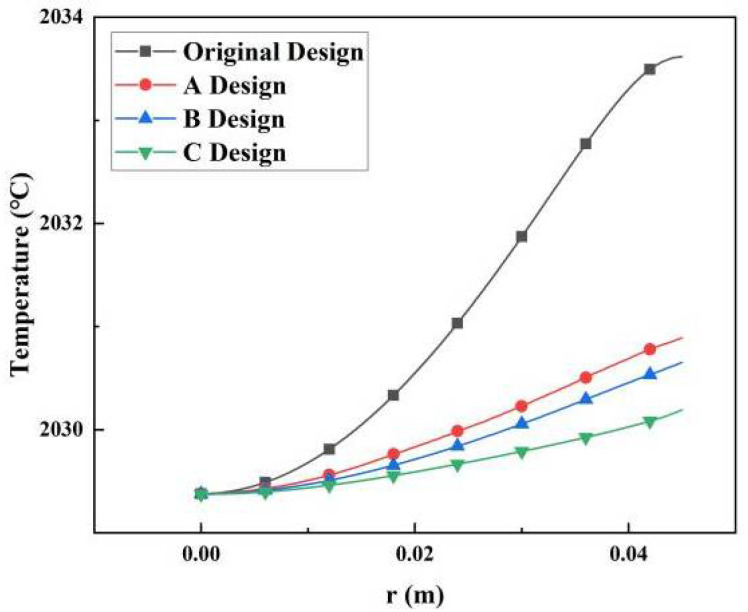
Radial temperature distributions of the AlN seed surface in different types of crucibles.

The radial temperature distributions of the AlN seed surface are shown in Figure 3. The tangent of temperature distribution (i.e., the temperature gradient) was reduced for all of the improved designs. The bottom fin created a uniform radial temperature distribution by blocking heat radiation from the crucible lid. As a result, the temperature gradient gradually decreased as the size of bottom fin gradually increased. 

### 3.2. Temperature Gradients in Radial and Axial Directions

**Figure 4:**
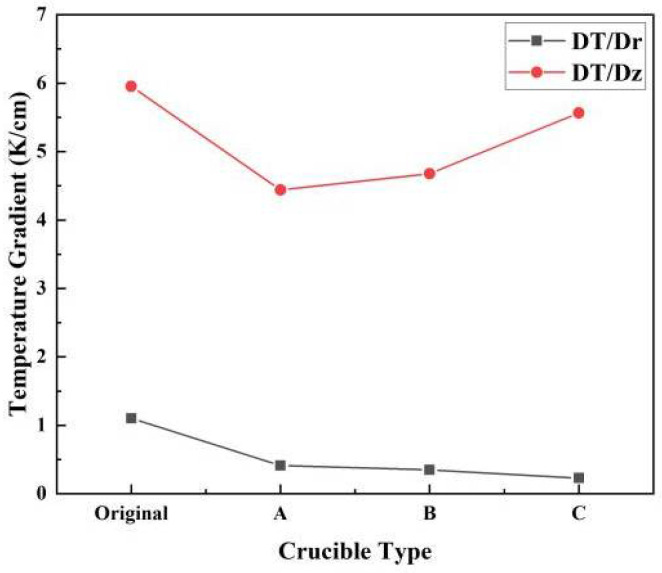
Temperature gradients of different types of crucibles (DT/Dz and DT/Dr represent the axial and radial temperature gradient, respectively).

Figure 4 shows the temperature gradients of the original and three improved designs. Compared with the original crucible, the addition of the tungsten sink reduced both the axial and radial temperature gradient. The reduction of the axial temperature gradient decreased the growth rate, which is not beneficial for industrial production [28]. However, because of the increased heat exchange area, the axial temperature gradient increased with an increase in the bottom fin diameter. To increase the axial temperature gradient with radial temperature uniformity, the bottom fin should cover the whole crucible lid. 

**Figure 5:**
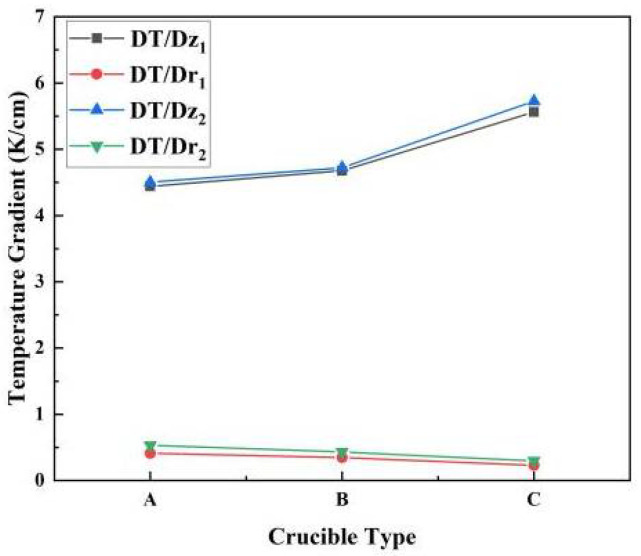
Comparison of temperature gradients in crucibles with different heat sinks. A, B and C designs represent designs with different bottom fin lengths; 1 and 2 represent tungsten sinks with and without the holder and top fin, respectively.

To further understand the functions of the top fin, we compared the temperature gradient difference between the tungsten sink designs with and without the top fin on the bottom fin (see Figure 5). Compared with the complete tungsten sink, the heat exchange of the crucible lid was weakened with only the bottom fin present. Removing the top fin resulted in a decrease of the axial temperature gradient and an increase in the radial temperature gradient. As the radial temperature gradient is the source of thermal stress, which causes the multiplication of dislocations and leads crystal growth to failure [29], it should be as small as possible. Therefore, the bottom fin combined with a holder and a top fin is beneficial to obtain a uniform radial temperature distribution with a less-affected axial temperature distribution. In addition, it can be concluded that enhancing the heat exchange of the crucible lid is an effective way to improve the quality of crystal growth.

### 3.3. Thermal Stress in the Seed

**Figure 6:**
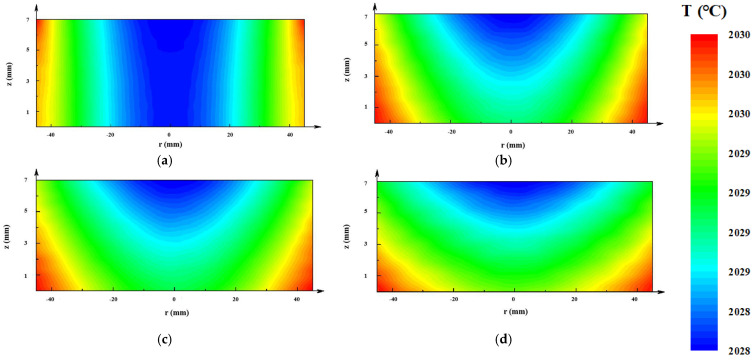
Temperature distributions of the AlN seed in different design crucibles: (**a**) original design; (**b**) A design; (**c**) B design; (**d**) C design.

Temperature distributions in the AlN seed are presented in Figure 6. Because of the radiation loss through the top window, the minimum temperature occurred in all designs at the center of the AlN seed top surfaces. The temperature distributions in the improved designs were greater than in the original design. 

**Figure 7:**
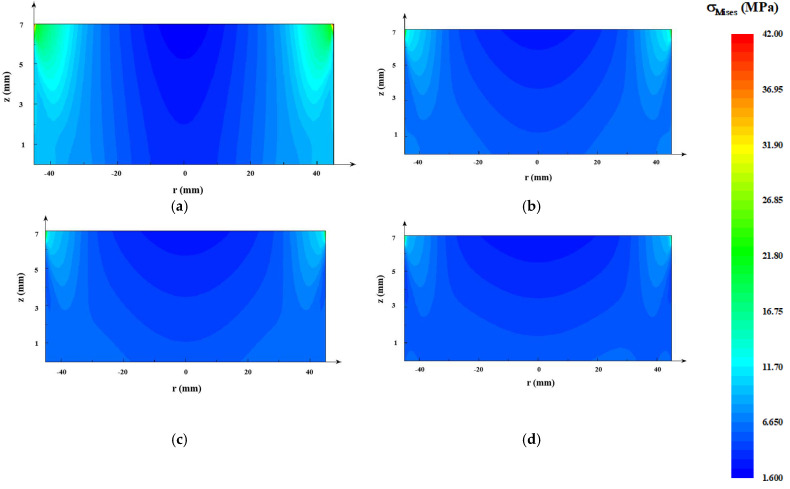
Thermal stress distributions of the AlN seed in crucibles with different designs: (**a**) original design; (**b**) A design; (**c**) B design; (**d**) C design.

The crystal did not contact the crucible wall. Thus, the thermal stress in the crystal was only caused by the non-uniformity of the temperature distribution. The von Mises stress is usually used as the resolved shear stress, which denotes the driving force for basal plane dislocation multiplication [30] (see Figure 7). All of the maximum thermal stresses occurred at the top periphery because of the boundary condition of the rigid top. The thermal stress in the improved designs decreased significantly compared with the original one. The minimal thermal stress appeared at the AlN crystal of the C design crucible. The thermal stress distribution also tended to be flat (see Figure 7a–d). 

### 3.4. Discussion

As can be seen in Figure 3, Figure 4, Figure 5, Figure 6 and Figure 7, the tungsten sink design was useful for large-size AlN single crystal growth. It can reduce the radial temperature gradient and thereby reduce the thermal stress. Thermal stress causes the multiplication of dislocations and results in crystal fragmentation. An effective tungsten sink design can not only improve radial temperature distribution, but also maintain a reasonable axial temperature gradient, which means that it may reduce dislocations in crystal growth and guarantee a normal growth rate. 

## 4. Conclusions

This study proposed the use of a tungsten sink on the top of the crucible during the sublimation growth of 45 mm AlN single crystals. Numerical experiments showed that the radial temperature gradient was reduced due to the homogenization effect of temperature by a tungsten sink. Because dislocations are one of the main problems in enlarging the size of AlN crystals, this simple tungsten sink method has great potential to grow large-size AlN ingots with reduced occurrence of cracking. 

## Figures and Tables

**Table 1:** Thermophysical properties of materials in the growth chamber.

	Thermal Conductivity (W/m·K)	Density (kg/m^3^)	Heat Capacity (J/kg·K)
Tungsten crucible	180	19,300	135
Insulation	0.5	170	2100
AlN powder	22.55	270.34	1172.7
AlN seed	320	3250	1197
